# A Label-Free Approach for Relative Spatial Quantitation of c-di-GMP in Microbial Biofilms

**DOI:** 10.1021/acs.analchem.3c04687

**Published:** 2024-05-16

**Authors:** Catherine S. McCaughey, Michael A. Trebino, Allyson McAtamney, Ruth Y. Isenberg, Mark J. Mandel, Fitnat H. Yildiz, Laura M. Sanchez

**Affiliations:** † Department of Chemistry and Biochemistry, University of California, Santa Cruz, Santa Cruz, California 95064, United States; ‡ Department of Microbiology and Environmental Toxicology, University of California, Santa Cruz, Santa Cruz, California 95064, United States; § Department of Medical Microbiology and Immunology, University of Wisconsin-Madison, Madison, Wisconsin 53706, United States; ∥ Microbiology Doctoral Training Program, University of Wisconsin-Madison, Madison, Wisconsin 53706, United States

## Abstract

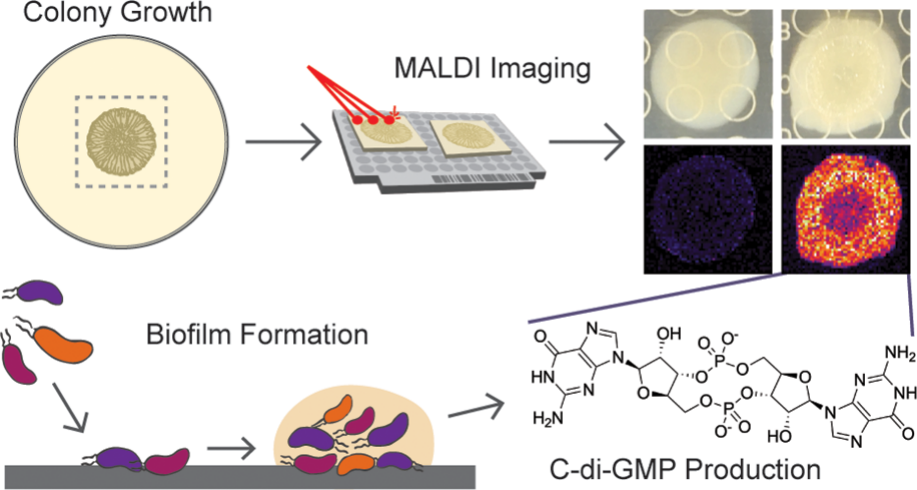

Microbial biofilms represent an important lifestyle for bacteria and are dynamic three-dimensional structures. Cyclic dimeric guanosine monophosphate (c-di-GMP) is a ubiquitous signaling molecule that is known to be tightly regulated with biofilm processes. While measurements of global levels of c-di-GMP have proven valuable toward understanding the genetic control of c-di-GMP production, there is a need for tools to observe the local changes of c-di-GMP production in biofilm processes. We have developed a label-free method for the direct detection of c-di-GMP in microbial colony biofilms using matrix-assisted laser desorption/ionization mass spectrometry imaging (MALDI-MSI). We applied this method to the enteric pathogen *Vibrio cholerae*, the marine symbiont *V. fischeri*, and the opportunistic pathogen *Pseudomonas aeruginosa* PA14 and detected spatial and temporal changes in c-di-GMP signal that accompanied genetic alterations in factors that synthesize and degrade the compound. We further demonstrated how this method can be simultaneously applied to detect additional metabolites of interest from a single sample.

## Introduction

Cyclic dimeric guanosine monophosphate (c-di-GMP) is a universally important second messenger that regulates exopolysaccharide production, virulence, antimicrobial tolerance, flagellar motility, cell morphology, and cell cycle control across nearly all bacterial phyla.^1−3^ C-di-GMP is synthesized by diguanylate cyclases (DGCs) and hydrolyzed by phosphodiesterases (PDEs). DGCs contain a GGDEF catalytic domain for cyclizing two molecules of guanosine triphosphate (GTP), while PDEs contain either an EAL or HD-GYP domain, which hydrolyze c-di-GMP to the linearized form (pGpG) or two molecules of guanosine monophosphate (GMP), respectively.^4^ The regulatory role of c-di-GMP is particularly important in biofilm-forming bacteria, as evidenced by the high number of DGC and PDE enzymes present in the genomes of biofilm-forming organisms such as *Pseudomonas aeruginosa*, *Vibrio cholerae*, and *Vibrio fischeri* including many enzymes with dual DGC/PDE activity.^1,5,6^

There are a variety of methods available to detect c-di-GMP in biological samples. LC–MS/MS analysis is currently the most accurate and sensitive method for detecting c-di-GMP, particularly in liquid extracts of nutrient broths, which provide a cumulative amount of c-di-GMP from diverse populations of bacterial cells. Fluorescent detection of c-di-GMP is also commonly used on bulk liquid samples and solid samples, and has many advantages in efficiency of detection and application engineered in vivo systems.^5^ Numerous innovative approaches for the detection of c-di-GMP using fluorescent biosensors have been recently developed including fluorescent reporter systems,^7,8^ riboswitch-based biosensors,^9,10^ FRET-based biosensors,^11^ and others.^12−14^ In general, fluorescent detection methods are applied to bulk samples but can also be used to detect the spatial and temporal dynamics of c-di-GMP signaling in biofilms.^7,15^ However, these fluorescence methods rely on engineered strains or reporters that do not allow for a facile extension to environmental isolates or emerging pathogens and are indirect measures of c-di-GMP levels.

Generally, high c-di-GMP levels induce the expression of biofilm-related genes, resulting in an overproduction of extracellular matrix and a wrinkled phenotype in surface-grown colonies, however, there are also inconsistencies in this model.^4,16,17^ Particularly in organisms harboring a large number of DGC and PDE domains, which control different c-di-GMP-dependent processes and effectors, it is often seen that deletion or overexpression of single DGC or PDE genes results in unexpected changes to the global c-di-GMP levels.^16^ Biofilms have heterogeneous structures and chemical gradients that change dynamically over time and influence the regulation of these DGC and PDE enzymes.^18,19^ MS imaging (MSI) is an analytical technique that allows for the spatial detection of small molecules (100–2000 Da) in biological samples, including tissues, whole organisms, and bacterial samples grown on a variety of substrates.^20^ MSI can achieve an image resolution as low as 10–20 μm and enables the robust detection of thousands of chemical species in a single experiment.^21^ This label-free technique has a significant advantage over fluorescent detection techniques in that both known and unknown chemical species can be correlated spatially with biofilm formation and c-di-GMP metabolism. At present, microbial MSI requires optimization for different microbial cultures and analyte classes.^21^

Here we present an MSI technique for the direct detection of c-di-GMP in bacterial biofilm colonies grown on solid agar media using matrix assisted laser desorption/ionization (MALDI) mass spectrometry imaging (MSI). We validated our MSI technique using a fluorescent riboswitch in *V. cholerae* and we further applied the MSI detection of c-di-GMP to the Hawaiian bobtail squid symbiont *Vibrio fischeri* and the medically relevant pathogen *P. aeruginosa* PA14. This technique is generally applicable to biofilm-forming bacterial species and has significant potential to generate biological hypotheses regarding inter-related metabolic pathways influencing biofilm formation and dispersal.

## Results

### Optimization of MALDI-MSI Parameters

**Figure 1:**
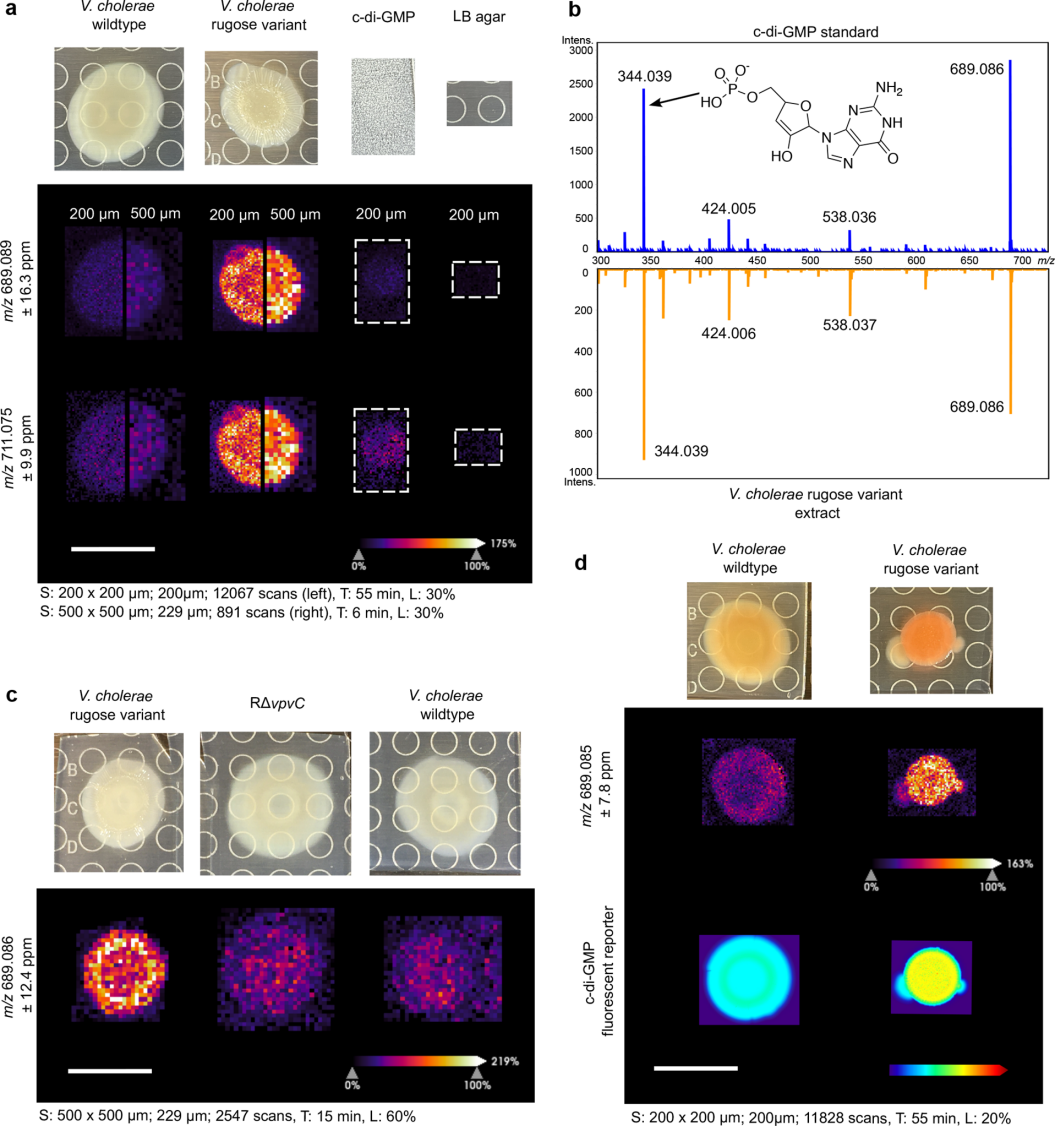
Spatial detection and validation of c-di-GMP in *V. cholerae* wildtype and rugose variant strains. (a) Ion images of the [M-H]^−^ (*m*/*z* 689.089) and [M-2H+Na]^−^(*m*/*z* 711.075) adducts of c-di-GMP in *V. cholerae* wildtype and rugose variant colonies. Data were acquired at both 200 and 500 μm raster to compare image resolution and sensitivity. An authentic standard of c-di-GMP (5 μL, 100 nM) was spotted on a sample of LB agar for comparison. (b) MALDI-MS/MS spectrum of c-di-GMP compared to an extract of a *V. cholerae* rugose variant colony grown on LB agar. (c) Ion images of c-di-GMP in *V. cholerae* wildtype, rugose variant, and a rugose variant Δ*vpvC* (*R*Δ*vpvC*). (d) Ion images of c-di-GMP in *V. cholerae* wildtype and the rugose variant compared to the c-di-GMP specific reporter. Abundance of c-di-GMP is represented by heat maps showing the relative TurboRFP fluorescent signal in the same bacterial colonies. Spot raster; size; scan number (S), acquisition time (T), and laser power (L) shown for each MSI experiment. All scales bars represent 1 cm.

Initially, we sought to test both positive mode and negative mode matrices for their ability to ionize c-di-GMP. For positive mode imaging, a 1:1 mixture of α-cyano-4-hydroxycinnamic acid (CHCA) and 2,5-dihydroxybenzoic acid (DHB) matrices were applied using a sieve method for bacterial colonies grown on agar media.^21^ We tested two other MALDI matrices that are known to facilitate ionization of nucleotides and nucleosides, 2′,4′,6′-trihydroxyacetophenone (THAP) and 3-hydroxypicolinic acid (HPA),^22,23^ in addition to CHCA and DHB in negative ionization mode. We tested the ionization of a c-di-GMP chemical standard with a dried drop method using a 1:1 CHCA:DHB mixture, as well as CHCA, THAP, and HPA each as the sole matrix in both positive and negative mode. The positive ionization mode did not provide adequate c-di-GMP ionization regardless of the MALDI matrix used. This is likely due to the fact that typical acidic additives such as TFA cannot be added to matrices applied using the dry sieve matrix application method.^21^ In negative mode, we found that c-di-GMP ionizes well when CHCA is used alone or in combination with DHB, while the THAP and HPA matrices did not induce sufficient ionization at low c-di-GMP concentrations (Figure S1). We opted to move forward using 1:1 CHCA:DHB for MSI analysis in negative mode, as it provided sufficient ionization for c-di-GMP (Figure S2), and was already optimized for the sieving method of matrix application and sample drying for MSI. These sample conditions may also be used on bacterial samples that are dried and prepared using a spray method and further modifications can be made such as using CHCA alone, or using acidic additives to enhance positive mode ionization of c-di-GMP. We tested both 500 and 200 μm raster widths on the bacterial biofilm colonies initially to determine the proper resolution for comparing the spatial distribution of c-di-GMP (Figure 1a). The 200 μm raster width provided a clearer image that aligned well with the biofilm structures observed in the optical images, so we moved forward using the 200 μm raster width in all subsequent experiments. While these raster widths work well for the agar-grown biofilm colonies analyzed in this study, this parameter can easily be adjusted for other preparations of biofilms (i.e., cryosectioning) as well as host tissue analysis to achieve higher resolution images of in situ biofilm infections.

### Spatial Detection of c-di-GMP in *V. cholerae* Biofilm Colonies

As a proof of concept, we used two model *V. cholerae* El Tor strains to compare c-di-GMP production, one of which is well-established as an overproducer of c-di-GMP. The *V. cholerae* O1 El Tor A1552 wildtype strain produces a smooth phenotype when grown on LB agar, while the *V. cholerae* O1 El Tor A1552 rugose variant produces a wrinkled phenotype on LB agar due to an excess of biofilm production (Table S1). The *V. cholerae* rugose variant used here has been well-established as a biofilm producer due to the activity of the DGC VpvC, which has a tryptophan to arginine amino acid residue change as the result of a single nucleotide polymorphism compared to the wildtype strain, rendering it hyperactive.^24,25^

Our initial results using wildtype *V. cholerae* and the rugose variant showed that c-di-GMP can be detected as both *m*/*z* 689.089 [M-H]^−^ and *m*/*z* 711.075 [M+Na-2H]^−^ (Figure 1a). Both adduct ions of c-di-GMP show related distributions in the ion images of the wildtype and rugose variant *V. cholerae* strains, and the presence of these adducts was confirmed by the use of a c-di-GMP chemical standard applied by the dried droplet method to a piece of LB agar which had been prepared for MSI using the sieve method with 1:1 CHCA:DHB (Figure 1a). We further confirmed the identity of c-di-GMP using MS/MS fragmentation on dried droplet samples of a c-di-GMP chemical standard compared to an extract of the *V. cholerae* rugose variant biofilm colony (Figure 1b). When comparing the spatial c-di-GMP distribution between the wildtype and rugose variant, the rugose variant showed higher concentrations of c-di-GMP along the edge of the colony, whereas the wildtype *V. cholerae* produced c-di-GMP more diffusely throughout the colony (Figure 1a). The 200 μm raster width better aligned with the rugosity observed in the optical images of the *V. cholerae* rugose variant, while the wildtype did not have distinct spatial differences in c-di-GMP detection at either 200 or 500 μm raster widths (Figure 1a). We further analyzed a *V. cholerae* rugose variant lacking the dominant DGC, VpvC, using MSI (RΔ*vpvC*, Figure 1c). The RΔ*vpvC* mutant had a smooth colony morphology, which was reflected in the change in both intensity and distribution of c-di-GMP detected via MSI (Figure 1c).

### Orthogonal Validation of c-di-GMP Detection Using a Riboswitch Biosensor

To validate the spatial distributions we observed with MSI, we used an orthogonal fluorescent riboswitch biosensor for c-di-GMP detection.^9,26^ We grew strains on thin agar to directly compare growth conditions across both methodologies, imaged the colonies using a fluorescent microscope, then excised the same bacterial colonies for MALDI–MSI analysis. To measure c-di-GMP in live cells prior to MSI, we used a dual fluorescent c-di-GMP specific reporter that we have previously validated to accurately measure changes in c-di-GMP.^26^ The fluorescent protein AmCyan encoded in this biosensor is produced constitutively and is used to normalize expression of TurboRFP, which is regulated by two c-di-GMP riboswitches to report intracellular c-di-GMP levels. In this reporter, we introduced an AAV degron to TurboRFP to improve spatial measurement of c-di-GMP levels. Here we represent c-di-GMP abundance within spot biofilms as a heat map of TurboRFP fluorescent intensity throughout the biofilm; AmCyan fluorescence was comparable between strains. The *V. cholerae* biofilm colonies expressing the fluorescent biosensor in Figures 1d and S4 appear more red in the optical images compared to the unmodified *V. cholerae* rugose variant due to the expression of the biosensor.

The corresponding images shown in Figure 1d indicate that both the MSI and fluorescent biosensor detection methods can accurately detect differences between the low and high c-di-GMP producing strains. The agreement between the resulting images highlights that lateral diffusion is not a concern for the MSI experiments for c-di-GMP. However, the MSI images afforded a higher resolution in the *V. cholerae* rugose variant colonies and increased sensitivity in the wildtype *V. cholerae* colonies (Figures 1 and S4).

### Production of c-di-GMP in Rugose and Smooth *V. cholerae* Colonies Varies over Time

**Figure 2:**
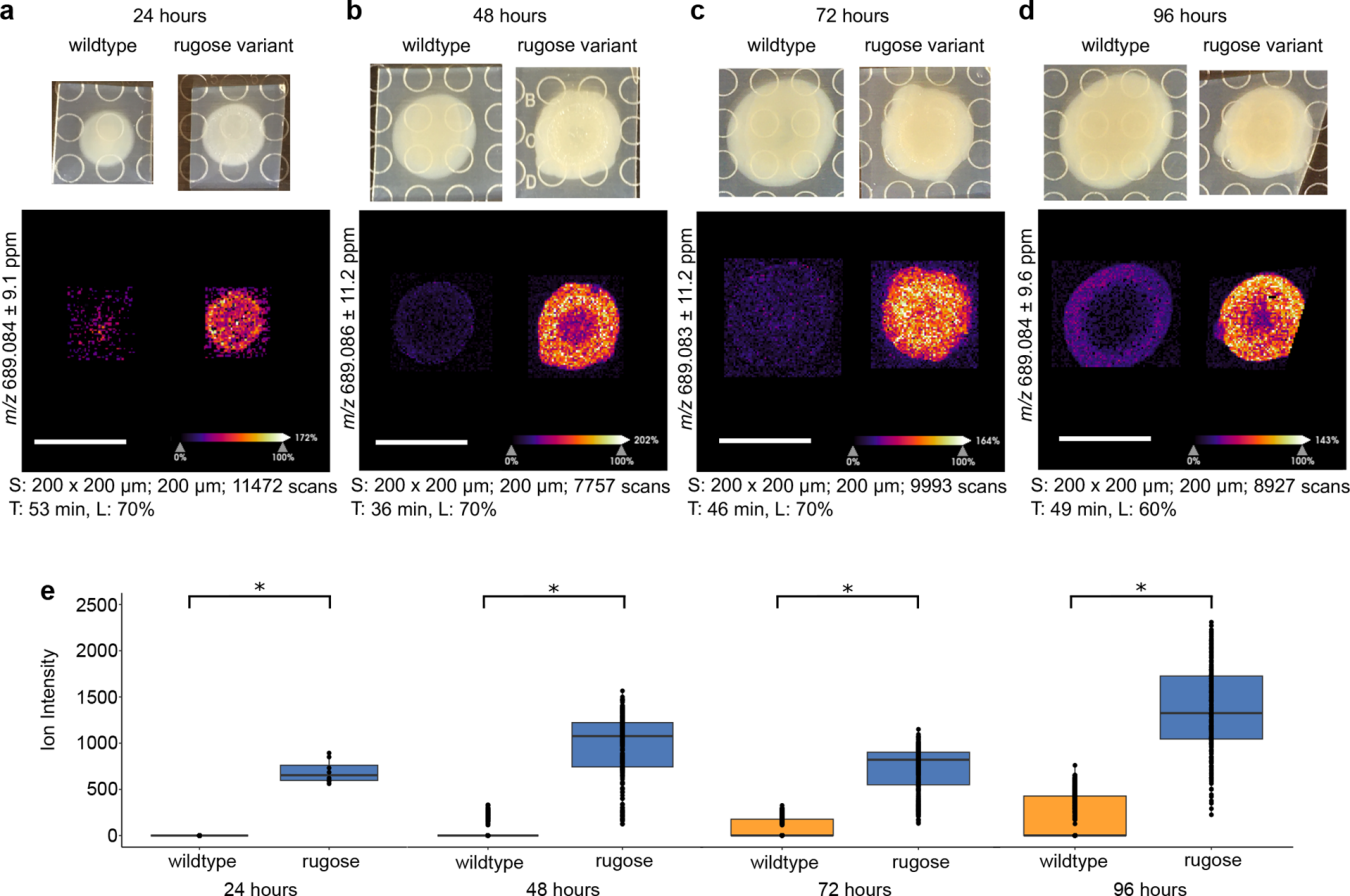
Comparison of c-di-GMP spatial distribution in *V. cholerae* colonies over time. Ion images and boxplots comparing ion intensity for *V. cholerae* wildtype and rugose variant strains after (a) 24 h, (b) 48 h, (c) 72 h, and (d) 96 h of growth. Spot raster; size; scan number (S), acquisition time (T), and laser power (L) shown for each MSI experiment. All scale bars represent 1 cm. (e) Box plots comparing ion intensity between *V. cholerae* wildtype and rugose variants grown on each day. The box plots are shown on a single graph, however the data from each time point were acquired as separate experiments. The line within each box plot represents the median, the edges of the box plots represent the upper and lower ends of the interquartile range and the whiskers represent the minimum and maximum quartiles. Outliers are shown beyond the whiskers of the box plots. Asterisks indicate statistical significance (*p* < 0.001) in a Wilcox Rank Sum test which was calculated using the SciLs MSI analysis software.

We sought to measure c-di-GMP levels over time in *V. cholerae* strains to observe how spatial changes in c-di-GMP production develop in wildtype *V. cholerae* and the rugose variant. All biofilm colonies were inoculated at the same time and three colonies each from the wildtype and rugose variant of *V. cholerae* were analyzed by MSI every 24 h for 4 days. At each time point, the rugose variant colonies produced more c-di-GMP than the wildtype colonies (Figure 2). We plotted the ion intensity of the c-di-GMP ion *m*/*z* 689.089 across all raster points in the *V. cholerae* wildtype and rugose variant colonies as boxplots and applied a Wilcox Rank Sum Test using the SCiLS MSI analysis software (Bruker Daltonics) as a statistical analysis to compare the overall ion intensity between the wildtype and rugose variants at each time point (Methods). This analysis demonstrated that the c-di-GMP signal was significantly higher (*p* < 0.001) in the rugose variant compared to the wildtype strain at all four time points (Figure 2), and validates previous bulk LC-MS/MS measurements of c-di-GMP in liquid cultures of these two strains.^27^ This difference was most apparent at 24 h, where c-di-GMP was barely detectable in the wildtype *V. cholerae* biofilm colonies but clearly detectable throughout the rugose variant colonies. At 24 h, the rugose variant colonies had just begun to wrinkle at the edges due to biofilm production, and the presence of c-di-GMP in the MSI ions images aligns well with this phenotype (Figures 2a and S5a).

The c-di-GMP levels in the wildtype *V. cholerae* biofilms remained low until 96 h and appeared with a distinct spatial distribution around the edge of the colony (Figures 2d and S5d). While the wildtype *V. cholerae* strain used here does not typically show any of the wrinkling phenotype associated with excess production of biofilm matrix, our results indicate that c-di-GMP is still produced in a spatially and temporally distinct manner in this strain. The rugose variant produced a much higher c-di-GMP signal throughout all of the time points, which also varied spatially with colony wrinkling. The rugose variant colonies formed a wrinkled ridge around the center of the colony, creating a visually distinct inner and outer colony starting at 48 h and persisting until 96 h (Figures 2 and S5). The c-di-GMP signal detected at these time points aligned with the inner ridge of the colony, either with a higher signal at the edges as in the 48 and 96 h time points (Figure 2b,d), or a higher signal in the center of the colony as in the 72 h time point (Figure 2c). These minor but observable differences among the sampled time points and biological replicates corroborate what is known about the dynamic control of c-di-GMP through complex signal transduction pathways and a multitude of differentially expressed DGCs and PDEs. Small regions of high c-di-GMP intensity within a microbial biofilm, such as in the 96 h *V. cholerae* rugose variant replicate in Figure S5d, clearly highlights the importance of understanding the spatial dynamics of c-di-GMP production. While LC-MS/MS provides a more precise bulk concentration of c-di-GMP, MSI can illuminate idiosyncrasies and nuanced changes in the spatial control of c-di-GMP.

### Imaging c-di-GMP in the Marine Bobtail Squid Symbiont *Vibrio fischeri*

**Figure 3:**
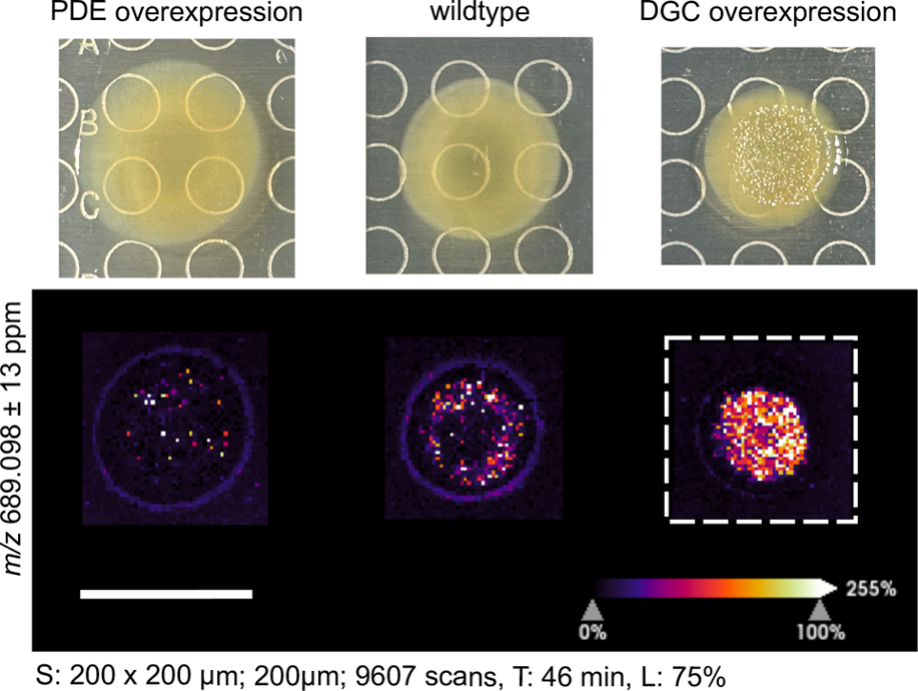
Comparison of c-di-GMP spatial distribution in *V. fischeri* colonies. The low c-di-GMP (PDE overexpression) and high c-di-GMP (DGC overexpression) strains contain a plasmid with an inducible promoter for the overexpression of the PDE VF_0087 and DGC MifA, respectively. The wildtype strain contains the vector control only. Spot raster; size; scan number (S), acquisition time (T), and laser power (L) shown for each MSI experiment. All scale bars represent 1 cm.

*V. fischeri*, another biofilm-producing relative in the *Vibrionaceae*, is the sole light organ symbiont of *Euprymna scolopes* (Hawaiian bobtail squid). The *V. fischeri* and *E. scolopes* relationship is a well-established model symbiosis that requires biofilm production by *V. fischeri* and for which c-di-GMP influences colonization behaviors.^6,28−31^ The symbiotic biofilm produced by *V. fischeri* is regulated differently from *V. cholerae* biofilm production.^29,32,33^ Cellulose production by *V. fischeri* is regulated by the c-di-GMP-responsive transcriptional regulator VpsR, a homologue of the *V. cholerae* master regulator of biofilm.^30,34−36^ This difference in biofilm regulation highlights the complexity and importance of studying the relative biological influence of c-di-GMP-mediated biofilm production in different contexts. To further validate MSI as an effective detection method for c-di-GMP, we applied well-established genetic manipulations to enzymes in the *V. fischeri* c-di-GMP pathway (Table S1).^37,38^ These two genetic alterations in the c-di-GMP pathway result in opposite c-di-GMP phenotypes when grown on agar,^39^ so we focused on the following *V. fischeri* ES114 derivatives to perform these studies: (i) a strain overexpressing *V. fischeri* PDE VF_0087^40^ from an IPTG-inducible vector and has smooth colony morphology (“PDE overexpression”; i.e., ES114/pEVS143-VF_0087), (ii) wildtype ES114 strain with the empty vector (“WT”; i.e., ES114/pRYI039), and (iii) a strain overexpressing *V. fischeri* DGC MifA^37^ that exhibits a wrinkled morphology (“DGC overexpression”; i.e., ES114/pEVS143-MifA) (Table S1, Figure 3). We have shown previously that an analogous gradient of strains can be used to detect other compounds that are upregulated in biofilm colonies, and here we show that these methods can be expanded to other compounds and genetic contexts.^41^

Using MSI we determined that the spatial distribution of c-di-GMP in *V. fischeri* colonies correlated to the wrinkles within these colonies, and the highest intensity signal across colonies assessed was associated with the DGC overexpression strain (Figures 3 and S6). Validating the strains used, the lowest intensity signal corresponded to the PDE overexpression strain, while a small amount of c-di-GMP was present in the wildtype (Figure 3). Similarly to *V. cholerae*, higher c-di-GMP levels were detected throughout the DGC overexpression colony, whereas lower levels of c-di-GMP were detected near the edges of the wildtype and PDE overexpression strains (Figure 3). To further validate these observations, we performed segmentation analysis to better understand the relationship between metabolite production colocalized to *V. fischeri* colony biofilms versus the edges of colonies (Figure S7). This analysis resulted in DGC overexpression colony-associated features clustering separately from features near the edge of the colony, suggesting unique metabolite production spatially correlates with increased biofilm production.

### Integration of Untargeted Metabolomics with c-di-GMP Imaging in *P. aeruginosa* PA14

**Figure 4:**
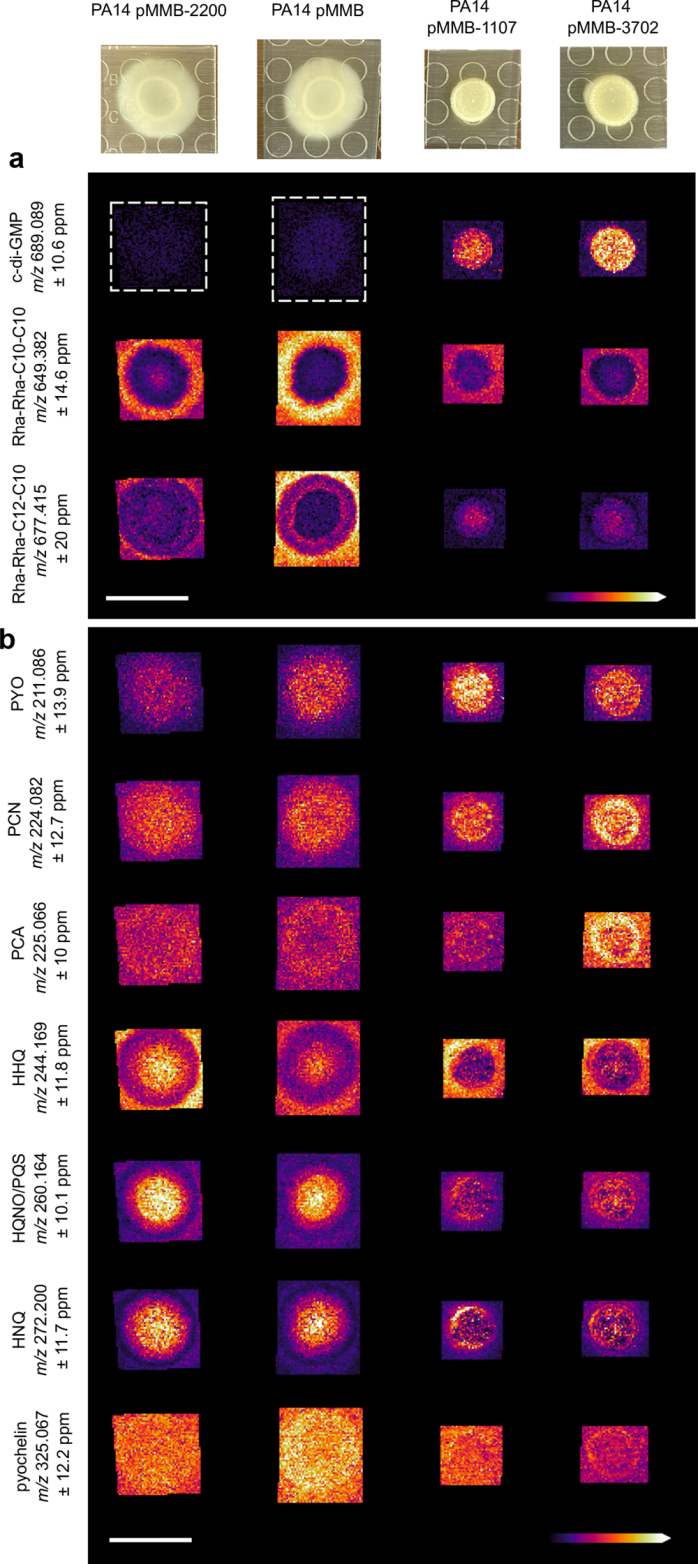
Ion images of c-di-GMP and other putatively identified metabolites in *P. aeruginosa* PA14. Ion images of metabolites detected in (a) negative mode ionization and (b) positive mode ionization. The following compound abbreviations are used: pyocyanin (PYO), phenazine-1-carboxamide (PCN), phenazine-1-carboxylic acid (PCA), Pseudomonas quinolone signal (PQS), 4-hydroxy-2-heptyquinoline-N-oxide (HQNO), 2-heptyl-4-quinolone (HHQ), and 4-hydroxy-2-nonylquinoline (HNQ). Table 1 shows the ppm error for all putatively identified compounds. Spot raster; size; scan number (S), acquisition time (T), and laser power (L) shown for each MSI experiment. All scale bars represent 1 cm.

*P. aeruginosa* is an important opportunistic pathogen and prolific producer of specialized metabolites, especially those related to virulence. Biofilm production, virulence, and specialized metabolism are intricately connected in *P. aeruginosa* through various signaling pathways.^42,43^ We have previously used MSI to study changes in metabolite production by *P. aeruginosa* PA14 due to the presence of an exogenous conjugated bile acid, taurolithocholic acid.^44^ Here we used MSI to analyze *P. aeruginosa* PA14 biofilms for both c-di-GMP production in negative mode ionization, and some common classes of important *Pseudomonas* metabolites in positive mode ionization (Figure 4). We applied this method to three *P. aeruginosa* PA14 strains, each containing an overexpression vector for a PDE (pMB-PA2200) or DGC (pMMB-PA1107, pMMB-PA3702) and an empty vector control (pMMB) (Table S1, Figures 4 and S8). The negative ionization results show a strong colony-associated signal for c-di-GMP in the DGC overexpression strains (PA1107, PA3702) compared to both the empty vector and PDE overexpression strain (PA2200) which show only background levels of c-di-GMP (Figures 4a and S8a,c). The strains used here were previously found to have differing virulence, biofilm phenotypes, and global levels of c-di-GMP due to the overexpression of specific DGCs or PDEs. However, the specific enzymatic contributions to the local pools of c-di-GMP could not be determined.^17^ Our results further validate the utility of our method to study local c-di-GMP production and spatial distribution in biofilm forming organisms under different genetic and environmental influences.

We then putatively identified other common specialized metabolites at the MS1 level in both negative and positive ionization modes from the same colony. Rhamnolipids are important biosurfactant molecules that are involved in mediating biofilm structure and dispersal.^45,46^ We detected two signals in negative mode that correspond to the rhamnolipids Rha-Rha–C10-C10 and Rha-Rha–C12-C10 (Figure 4a). Both rhamnolipids were detected at a higher intensity in the empty vector strain compared to all PDE and DGC overexpression strains (Figure 4a). This was expected as rhamnolipids are known to mediate cellular motility and are known to be anticorrelated with the production of phenazines such as pyocyanin (PYO) which are upregulated in biofilms.^47^ While we are not able to distinguish the isomeric rhamnolipid species using this method, we can identify distinct differences in the spatial distribution of different rhamnolipids in both low and high c-di-GMP conditions. Both the PDE overexpression strain, and the DGC overexpression strains had a decreased signal intensity for both rhamnolipids compared to the empty vector (Figure 4a), however we can also see that the Rha-Rha–C12-C10 rhamnolipid was detected more within the biofilm colonies in all conditions compared to the Rha-Rha-C10-C10 rhamnolipid which appears to be primarily secreted from the biofilm colony in all conditions (Figure 4a).

Phenazines are redox active molecules that are well-established as a microbial biofilm response to oxidative stress,^48^ and implicated in virulence in clinical isolates.^43^ We have previously shown how MSI can be used to interrogate changes in *P. aeruginosa* specialized metabolism due to both genetic mutations in the phz gene cluster and the presence of external chemical stimuli.^44^ Here our results show that phenazine production is slightly higher in the DGC overexpression strains compared to the empty vector control and the PDE overexpression strain (Figure 4b), but overall the phenazine production varies slightly between each strain. While the changes detected here are minor, the consistent detection of these important molecules may serve as a benchmark for understanding global changes to *P. aeruginosa* metabolism in different genetic and environmental contexts.

We also detected the quinolone family of quorum sensing molecules using positive mode ionization (Figure 4b). *Pseudomonas* quinolone signal (PQS), 4-hydroxy-2-heptyquinoline-N-oxide (HQNO), 2-heptyl-4-quinolone (HHQ), and 4-hydroxy-2-nonylquinoline (HNQ) are well established signaling molecules that we have previously identified in *P. aeruginosa* PA14.^44^ The isomeric species PQS and HQNO cannot be distinguished by our current MSI method (Figure 4b), although we have previously used orthogonal methods to demonstrate that PQS and HQNO are produced in different spatial distributions in PA14.^44^ The detected quinolones exhibited a distinct spatial distribution where HHQ had higher intensity around the edge of the biofilm colony compared to HQNQ/PQS and HNQ which showed the highest ion intensity only within the biofilm colony in all conditions (Figures 4b and S8b,d).

Pyochelin is a siderophore which is also implicated in virulence of *P. aeruginosa*. We have previously shown how pyochelin production is mediated by exposure to exogenous compounds such as bile acids.^44^ We found that the pyochelin signal was highest in the empty vector and the PDE overexpression strain, and lowest in the DGC overexpression strain pMMB-PA3702 (Figures 4b and S8b,d).

## Discussion

The signaling molecule c-di-GMP plays an important role in the complex coordination of bacterial metabolism throughout biofilm formation, maintenance, and dispersal. The accurate and sensitive detection of c-di-GMP in different contexts will inform how local and temporal changes in c-di-GMP concentrations control biofilm physiology. While many fluorescence-based techniques are in active development and expansion, we present a label-free MSI method for detecting c-di-GMP spatially in different organisms and environmental contexts. Here we show how this method can be applied to genetic mutants and natural isolates from both pathogenic and symbiotic contexts to compare spatial changes in local pools of c-di-GMP. All of the model microorganisms presented here have genetic modifications specifically related to their c-di-GMP production. Biological turnover of c-di-GMP is complex and is likely controlled by local environmental sensing to improve adaptability and flexibility throughout the infection process. These local fluctuations in c-di-GMP production that influence biofilm phenotypes are relevant in both medical and environmental contexts. There is a need to interrogate the relative influence of c-di-GMP in microenvironments both via genetic and environmental manipulations, but also in host tissue contexts.

Because c-di-GMP signaling in biofilm control plays a significant biological role in so many diverse environments, the translation of this method to host tissues is not trivial. Using the *E. scolopes* and *V. fischeri* model system, we have previously shown how MSI can be optimized on agar grown biofilms for further application to host tissues.^41^ The translation of our methodology to other host tissues such as *V. cholerae* infected intestines and *P. aeruginosa* lung or skin infections requires dedicated sample preparation optimization for each host tissue system that is analyzed. We provide a foundation for MALDI matrix selection and MSI parameters for detecting c-di-GMP in bacterial biofilms. Different types of host tissues and infection models will inherently contain differing cell densities and metabolic activity but our method demonstrates that the detection of high c-di-GMP producing aggregates may be facilitated by MALDI-MSI. Fluorescent probes for the identification of bacteria can also be applied for image coregistration, in addition to other multimodal imaging techniques. In order to verify the presence of c-di-GMP in any biological sample a chemical standard can be applied by spotting onto a tissue or combining with tissue homogenates to account for changes to ionization due to the complex chemical background of many biological tissues.

*P. aeruginosa* is well-studied for its arsenal of small metabolite virulence factors, and we have highlighted some of the commonly studied compounds that are known to influence *P. aeruginosa* virulence and biofilm phenotypes.^42,44,49^ Our MALDI-MSI method allows for the simultaneous detection of c-di-GMP as well as other specialized metabolites in both positive and negative ionization mode from a single sample by repeated laser irradiation in two experiments (Figure 4). While the global levels of c-di-GMP have been well-studied in relation to genetic and environmental manipulation, these measurements do not always correlate with the phenotypic alterations observed in different DGC or PDE mutants.^17^ The local contribution of different DGC and PDE enzymes is likely to be a major influence in the altered biofilm phenotypes that are observed.

## Conclusions

MALDI-MSI detection of c-di-GMP in bacterial colonies allows for the efficient observation of local changes to specialized metabolite pathways in correlation with changes in c-di-GMP levels. The versatility of this method allows for the spatial correlation of c-di-GMP with various specialized metabolites in genetically engineered strains as well as clinical isolates and has the potential to be optimized for the direct application to host tissues.

## Methods

### MALDI–MSI Analysis of Bacterial Colonies

The desiccated bacterial colonies and agar controls were then analyzed using a MALDI mass spectrometer (Bruker timsTOF fleX qTOF mass spectrometer). Imaging mass spectrometry data was acquired using timsControl v 4.1 and flexImaging software v. 7.2. The data were collected using the mass range 300–800 Da in negative mode and 100–1500 Da in positive mode. Data were acquired at 200 μm (200 × 200 μm laser size) or 500 μm (229 × 229 μm laser size) spatial resolution using the M5 defocus laser setting. Each raster point was acquired using 200 laser shots at 1000 Hz in all experiments. Other details regarding the acquisition parameters are indicated in each figure using the “SMART” standardized nomenclature.^50^ Step size, spot size, and scan number (S), molecular identification (M), annotations (A), mass resolution (R), time of acquisition (T) are provided. All molecular identifications (M) were done to the MS1 level or using a commercial standard, all annotations (A) were targeted and no database searching was used and the mass resolution (R) in all experiments was 65,000 fwhm at *m*/*z* 1222. Because these three parameters were consistent throughout all experiments, we only indicate step and spot size (S), time of acquisition (T) and additionally, the laser power (L) used in each experiment on each figure. Table 1 indicates the MS1 level ppm error of the putatively identified compounds in Figures 4 and S8. The ppm range shown in each ion image represents the bin width used to generate the ion image. The mass spectrometer was calibrated using red phosphorus as a calibrant. All raw data are available in MassIVE (massive.ucsd.edu) under the doi:10.25345/C5RV0DB1W.

**Table 1:** Mass Error for Detected *P. aeruginosa* Metabolites

compound	adduct	theoretical *m*/*z*	measured *m*/*z*	ppm error
Rha-Rha–C10-C10	[M-H]-	649.3804	649.3821	2.62
Rha-Rha–C12-C10	[M-H]-	677.4127	677.4147	2.95
PYO	[M + H]+	211.0866	211.0857	4.26
PCN	[M + H]+	224.0819	224.0810	4.02
PCA	[M + H]+	225.0659	225.0645	6.22
HHQ	[M + H]+	244.1696	244.1684	4.91
HQNQ/PQS	[M + H]+	260.1645	260.1637	3.07
HNQ	[M + H]+	272.2009	272.1994	5.51
pyochelin	[M + H]+	325.0675	325.0659	4.92

### Data Analysis

All ion images were generated using SCiLS lab software version 2023c pro (Bruker Daltonics). All ion images have hot spot removal applied and were normalized using a total ion chromatogram (TIC) normalization in SCiLS. The boxplot data were generated by exporting the sample regions from SCiLS for import into Metaboscape v. 2022b (Bruker Daltonics). Peak picking and thresholding was performed in metaboscape and a csv file was exported containing all of the mass spectra data for the *m*/*z* feature corresponding to the [M-H]^−^ ion of c-di-GMP (*m*/*z* 689.09). Boxplots were generated in R using the integrated ion intensity values of the [M-H]^−^ ion of c-di-GMP across the scans (raster points) in each ion image. The Wilcox Rank Sum test was run in SCiLS and all statistically significant results were determined to have a *p*-value <0.001 using SCiLS.

### Safety Note

Some of the microbes used in this research are considered biosafety level 2 and are opportunistic pathogens. We prepare samples in a biosafety cabinet to minimize exposure and once coated with matrix and dried, we consider the pathogens safe to handle, as they are no longer viable.

## Supporting Information Available

MALDI-MS spectra of the commercial c-di-GMP standard, replicate experimental data for all of the MSI experiments to support the figures depicted in the main text, segmentation map from data used in Figure 3, stability study of c-di-GMP (PDF)
