# Supplementary material for: A Label-Free Approach for Relative Spatial Quantitation of c-di-GMP in Microbial Biofilms
Source: Anal Chem. 2024 May 16;96(21):8308–16. doi: 10.1021/acs.analchem.3c04687 (PMC11140670; doi:10.1021/acs.analchem.3c04687)
Supplement: Supplementary file 1 — ac3c04687_si_001.pdf [file ac3c04687_si_001.pdf]

Supporting Information

A label-free approach for relative spatial quantitation of c-di-GMP in microbial biofilms

Catherine S. McCaughey,[1] Michael A. Trebino,[2] Allyson McAtamney,[1] Ruth Isenberg,[3,4,5] Mark J. Mandel,[3,4] Fitnat H. Yildiz,[2] Laura M. Sanchez[1,*]

[1]Department of Chemistry and Biochemistry, University of California, Santa Cruz, Santa Cruz, CA 95064
[2]Department of Microbiology and Environmental Toxicology, University of California, Santa Cruz, Santa Cruz, CA 95064
[3]Department of Medical Microbiology and Immunology, University of Wisconsin-Madison, Madison, WI 53706
[4]Microbiology Doctoral Training Program, University of Wisconsin-Madison, Madison, WI 53706
[5] Current Address: Department of Microbiology and Immunology, University of Minnesota Medical School, Minneapolis, MN 55455

*Corresponding author email: lmsanche@ucsc.edu

Materials and Methods. ………………………………………………………………… S2-3
Figure S1. MALDI-MS spectra of a commercial c-di-GMP standard…………………… S4
Figure S2. MALDI-MS and MS/MS spectra to test stability of c-di-GMP…………… S5
Figure S3. Replicates for *V. cholerae* wildtype and rugose variants...………………… S6
Figure S4. Replicate data for MALDI-MSI and fluorescent microscopy of *V. cholerae* S7
Figure S5. Comparison of c-di-GMP spatial distribution in *V. cholerae* over time…… S8-9
Figure S6. Replicate data for MALDI-MSI of *V. fischeri* …..………………………… S10
Figure S7. Segmentation data for MALDI-MSI of *V. fischeri*……………………………. S11
Figure S8. Replicate data for MALDI-MSI of *P. aeruginosa* PA14…………………... S12-13
Table S1. Bacterial strains, genotypes, and sources…………………………………… S14

Materials and Methods

Bacterial strains and growth conditions

All bacterial strains used in this study are described in Supplementary Table S1. All *V. cholerae* strains were grown in Bacto LB media (10g NaCl, 10 g tryptone, 5 g yeast extract, pH 7.5 in 1 L DI H2O). The *V. cholerae* (biosensor) strains and *P. aeruginosa* PA14 strains were grown in LB media containing 15 µg/mL gentamicin. *V. fischeri* was grown in LBS (LuriaBertani salt: 20 g NaCl, 10 g tryptone, 5 g yeast, 50 mL 1 M Tris, pH 7.0 in 1 L DI H_2O) agar with 100 µg/mL kanamycin.

All bacteria were plated on 1.5% agar media and grown overnight at 30 °C. A colony from the plate was then used to inoculate a 5 mL liquid culture of and grown overnight at 30 °C while shaking at 225 rpm. Overnight liquid cultures were normalized to an OD_{600} of 0.1 by diluting in sterile media and 5 µL of inoculum was spotted on thin agar plates (3 mL of agar in a 60 mm plate) and incubated for up to 96 h at 30 °C (*V. fischeri* was grown at room temperature). All solid and liquid bacterial cultures were grown in ambient air and humidity conditions. For *P. aeruginosa* PA14 strains, 100 µM IPTG was added to the thin agar plates for plasmid induction in the bacterial colonies that were analyzed by MALDI-MSI.

Colony sample preparation for MSI

We previously published a detailed protocol on the utilization of MALDI-MSI for the chemical analysis of bacterial biofilms,[1] which is briefly summarized here. Following 96 h of growth, colonies were excised from the agar plates using a razor blade and transferred to an MSP 96-target ground-steel target plate (Bruker Daltonics). Two additional pieces of sterile agar were excised and transferred to the target plate as controls. An optical image of the colonies on the target plate was taken prior to matrix application. A 53 µm stainless steel sieve (Hogentogler Inc.) was used to coat the steel target plate and colonies with MALDI matrix. The MALDI matrix used for the analysis was a 1:1 mixture of recrystallized δ-cyano-4-hydroxycinnamic acid (CHCA) and 2,5-dihydroxybenzoic acid (DHB) (Sigma). The plate was then placed in an oven at 40 °C for approximately 4 h or until the agar was fully desiccated. After 4 h, excess matrix was removed from the target plate and sample with a stream of air. A chemical

standard of c-di-GMP (1uL, 100nM) was spotted using a dried droplet method, onto one of the desiccated agar control samples. Another optical image was taken of the desiccated colonies on the target plate.

Bacterial colony extraction for MALDI-MS/MS

A standard dried droplet technique for spotting chemical samples for MALDI analysis involves mixing 1 μL of a dissolved sample with 1 μL of a saturated solution of MALDI matrix and spotting the mixture onto a MALDI target plate. This method was used for all chemical standards of c-di-GMP dissolved in water and mixed with a 1:1 mixture of CHA:DHB dissolved in 78:22 acetonitrile:H_2O.

In order to use MS/MS fragmentation to identify c-di-GMP in the bacterial biofilm produced by the *V. cholerae* rugose variant, we took a bacterial colony grown on thin agar for four days and scraped the entire colony into a saturated mixture of 1:1 CHCA:DHB in 1:1 methanol:H_2O. We then vortexed the sample, centrifuged (13,000 rpm, 2 min), and spotted this mixture directly onto the MALDI target plate for analysis. MALDI MS/MS data were acquired in negative ionization mode using a total of 1000 laser shots at 1000 Hz, selecting for *m/z* 689.0866 with a mass isolation window of 0.10 Da and a collision energy of 40 eV.

Microscopy for biosensor imaging

Colonies were prepared as previously described for MALDI-MS/MS analysis using the strain harboring the c-di-GMP specific biosensor. These were grown on 1.5% agar LB plates containing gentamicin and grown for 72 hours. Colonies were imaged using a Zeiss AxioCam HRm with a 1x/.025 NA lens on an Axiozoom V.16 stand. Amcyan was measured with an excitation of 470/40, a 495 dichroic mirror, and an emission of 525/50. TurboRFP was measured with an excitation of 550/25, a 570 dichroic mirror of 570, and an emission of 605/70. Acquisition parameters were identical between samples for comparison of fluorescent intensity. Images were analyzed using Zen Blue software. Quantification of c-di-GMP was then performed in parallel using MALDI-MS/MS.

Figure S1. MALDI-MS spectra of a commercial c-di-GMP standard crystallized with four different MALDI matrices, **a)** α-cyano-4-hydroxycinnamic acid (CHCA) alone, **b)** a 1:1 mixture of CHCA and dihydroxybenzoic acid (DHB), **c)** 2′,4′,6′-Trihydroxyacetophenone (THAP), and **d)** 3-hydroxypicolinic acid (HPA)

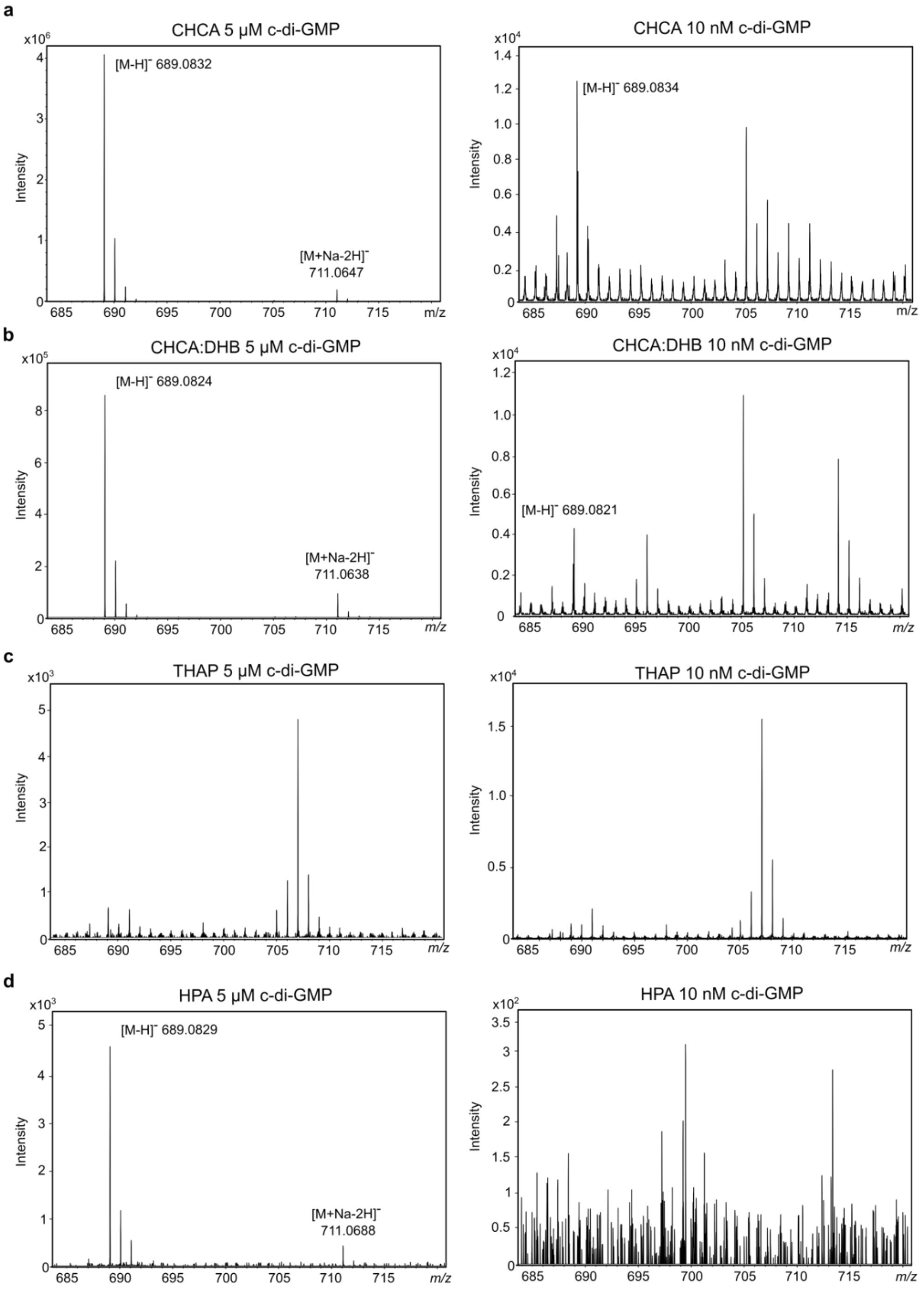

Figure S2. In order to test the stability of c-di-GMP, we spotted a commercial standard of c-di-GMP with the MALDI matrix and incubated this mixture 4 hours at 37 °C and freshly spotted the same mixture without incubation and measured both spots and also collected MS/MS data. Below, we provide MS and MS/MS spectra for a 1 μM solution of c-di-GMP standard. The blue spectra represent the standard spotted on a MALDI target plate with 1:1 CHCA:DHB and incubated for 4 hours at 37 °C, and the red spectra represent the same solution mixed with the same matrix just before MALDI analysis. We do not observe any degradation of the c-di-GMP standard, and we remain confident that there is minimal to no degradation in bacterial colonies during the drying process under the same temperature and time frame for sample preparation.

MS spectra

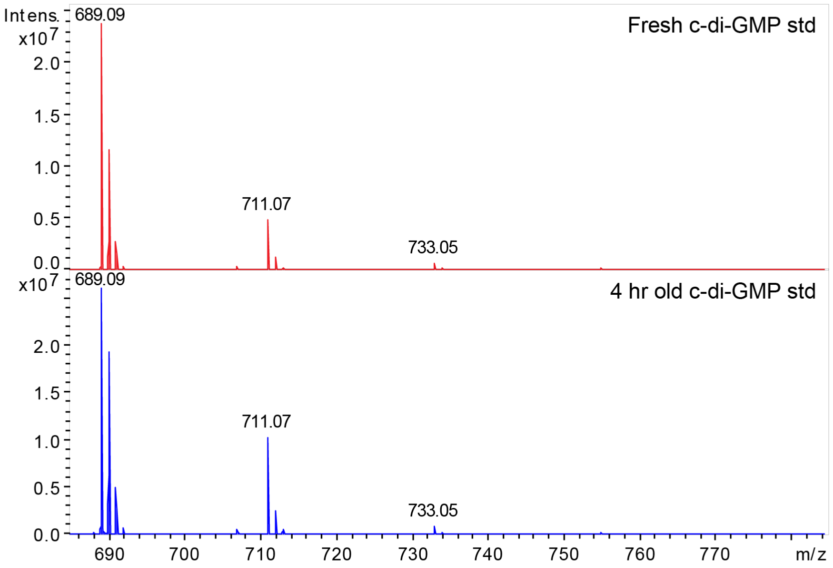

MS/MS spectra

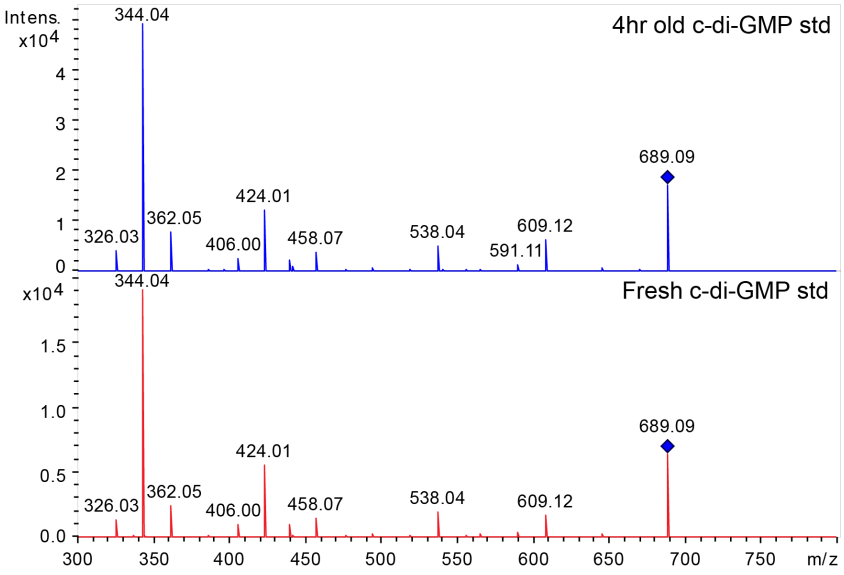

Figure S3. Biological replicate data for MALDI-MSI of *V. cholerae* in Figure 1. **a-b)** Ion images of replicates for *V. cholerae* wildtype and rugose variants from Fig. 1a. **c-d)** Ion images of replicates for *V. cholerae* wildtype, rugose variant, and *RΔvpvC* from Fig. 1c. Spot raster; size; scan number (S), acquisition time (T), and laser power (L) shown for each MSI experiment. All scale bars represent 1 cm.

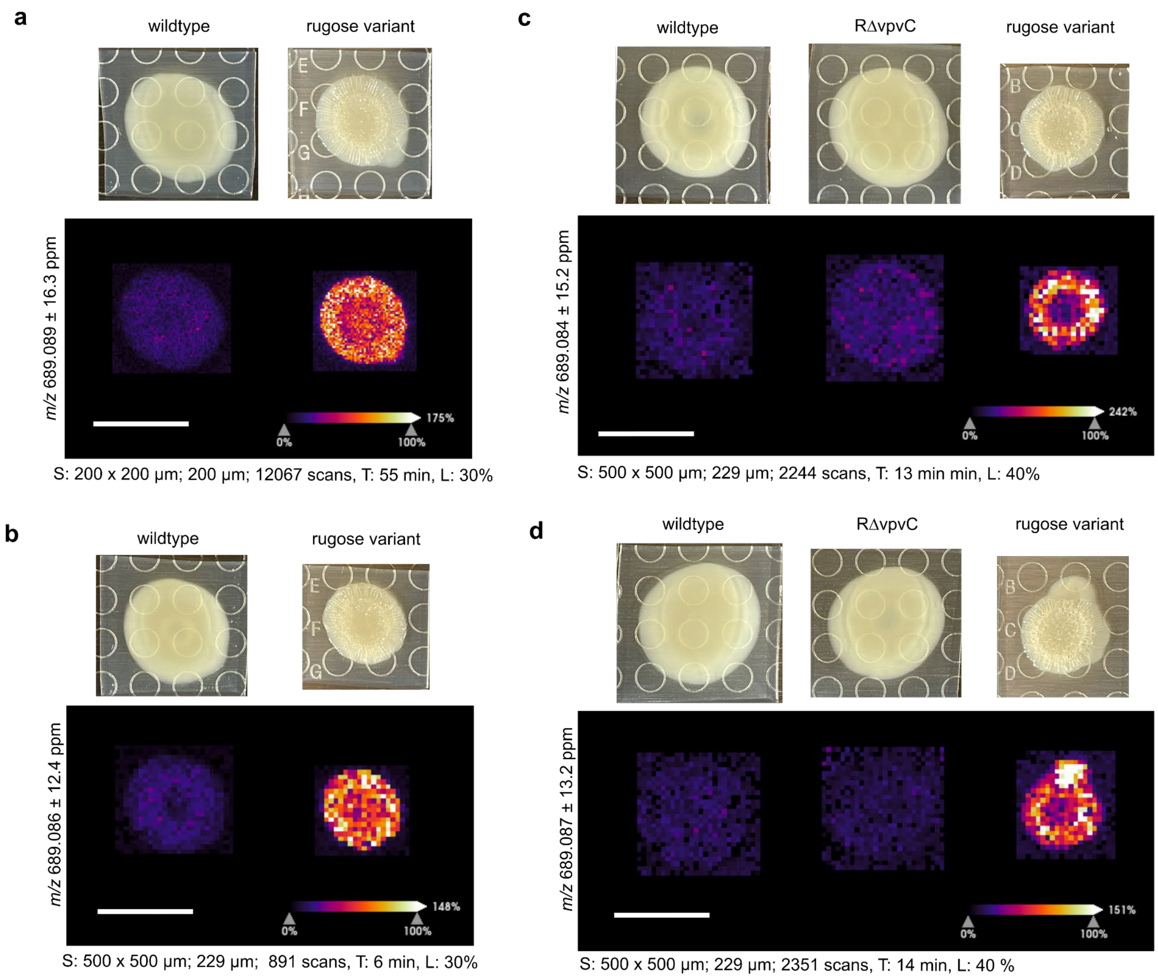

Figure S4. Biological replicate data for MALDI-MSI and fluorescent microscopy of *V. cholerae* in Figure 1d. Ion images of c-di-GMP in *V. cholerae* wildtype and the rugose variant compared to the c-di-GMP specific reporter. Abundance of c-di-GMP is represented by heat maps showing the relative TurboRFP fluorescent signal in the same bacterial colonies. Spot raster; size; scan number (S), acquisition time (T), and laser power (L) shown for each MSI experiment. All scale bars represent 1 cm.

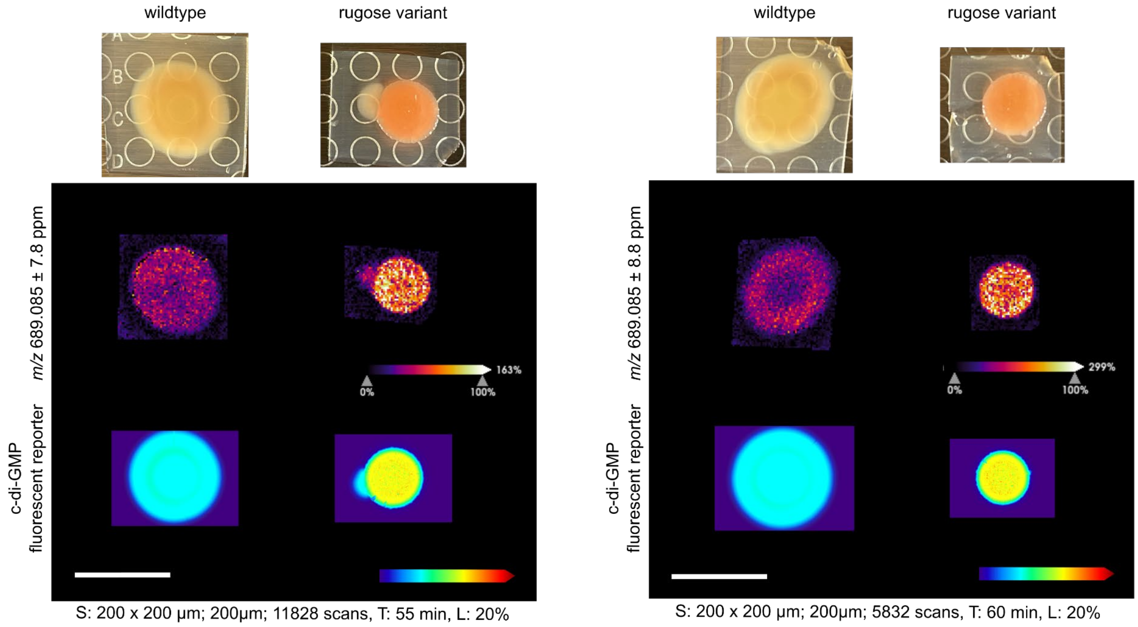

Figure S5. Biological replicate data for MALDI-MSI of *V. cholerae* in Figure 2. Comparison of c-di-GMP spatial distribution in *V. cholerae* colonies over time. Ion images for *V. cholerae* wildtype and rugose variant strains after **a)** 24 hours, **b)** 48 hours, **c)** 72 hours, and **d)** 96 hours of growth. Photos in d) show the biofilm colonies after MALDI matrix application and drying. Spot raster; size; scan number (S), acquisition time (T), and laser power (L) shown for each MSI experiment. All scale bars represent 1 cm.

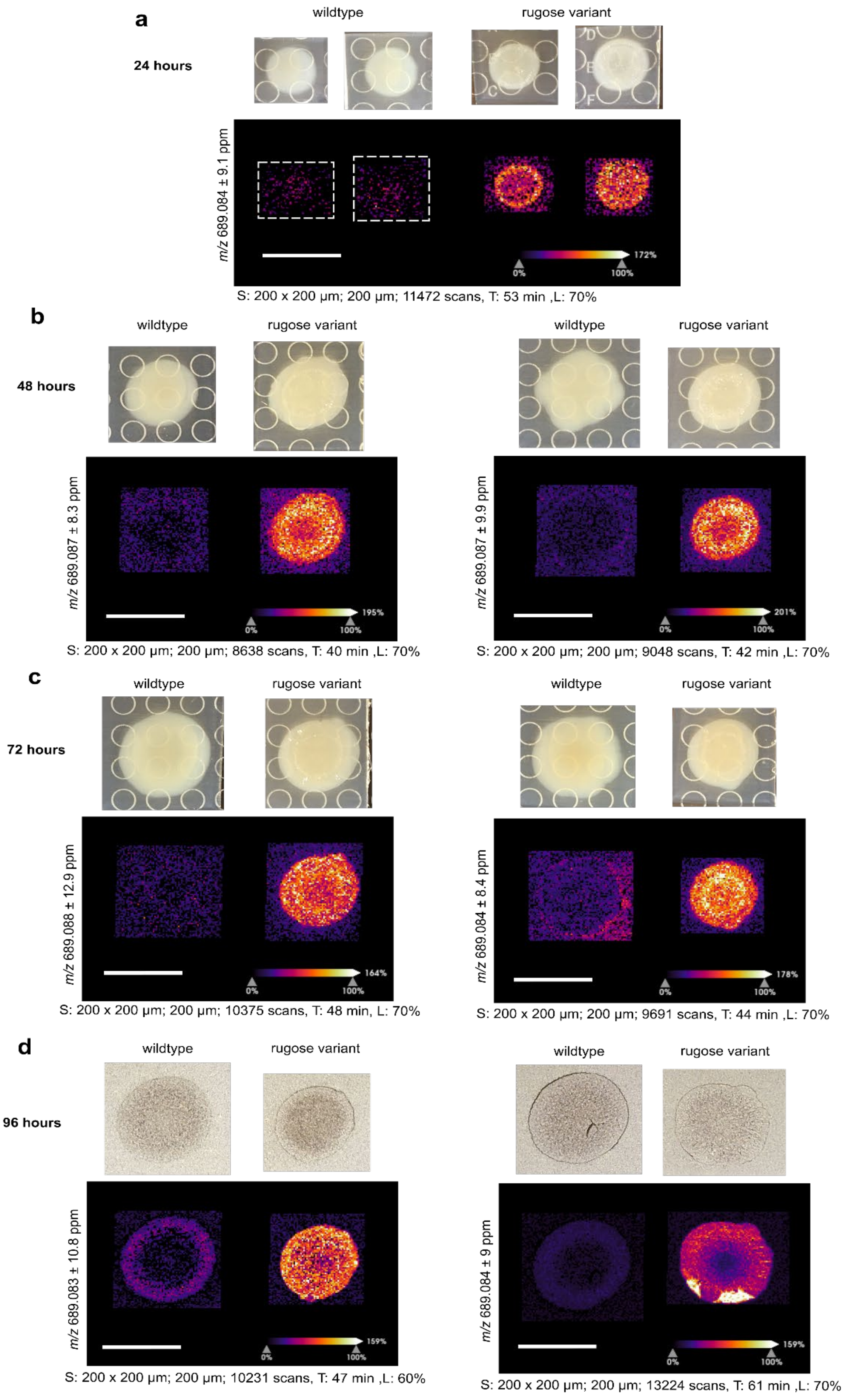
a
wildtype
rugose variant
24 hours
m/z 689.084 ± 9.1 ppm
172%
0%
100%
S: 200 x 200 µm; 200 µm; 11472 scans, T: 53 min ,L: 70%
b
wildtype
rugose variant
wildtype
rugose variant
48 hours
m/z 689.087 ± 8.3 ppm
195%
0%
100%
m/z 689.087 ± 9.9 ppm
201%
0%
100%
S: 200 x 200 µm; 200 µm; 8638 scans, T: 40 min ,L: 70%
S: 200 x 200 µm; 200 µm; 9048 scans, T: 42 min ,L: 70%
c
wildtype
rugose variant
wildtype
rugose variant
72 hours
m/z 689.088 ± 12.9 ppm
164%
0%
100%
m/z 689.084 ± 8.4 ppm
178%
0%
100%
S: 200 x 200 µm; 200 µm; 10375 scans, T: 48 min, L: 70%
S: 200 x 200 µm; 200 µm; 9691 scans, T: 44 min ,L: 70%
d
wildtype
rugose variant
wildtype
rugose variant
96 hours
m/z 689.083 ± 10.8 ppm
159%
0%
100%
m/z 689.084 ± 9 ppm
159%
0%
100%
S: 200 x 200 µm; 200 µm; 10231 scans, T: 47 min ,L: 60%
S: 200 x 200 µm; 200 µm; 13224 scans, T: 61 min ,L: 70%

Figure S6. Biological replicate data for MALDI-MSI of *V. fischeri* in Figure 3. Ion images of c-di-GMP in three strains of *V. fischeri.* The low c-di-GMP (PDE overexpression) and high c-di-GMP (DGC overexpression) strains contain a plasmid with an inducible promoter for the overexpression of the PDE VF_0087 and DGC MifA, respectively. The wildtype strain contains the vector control only. Spot raster; size; scan number (S), acquisition time (T), and laser power (L) shown for each MSI experiment. All scale bars represent 1 cm.

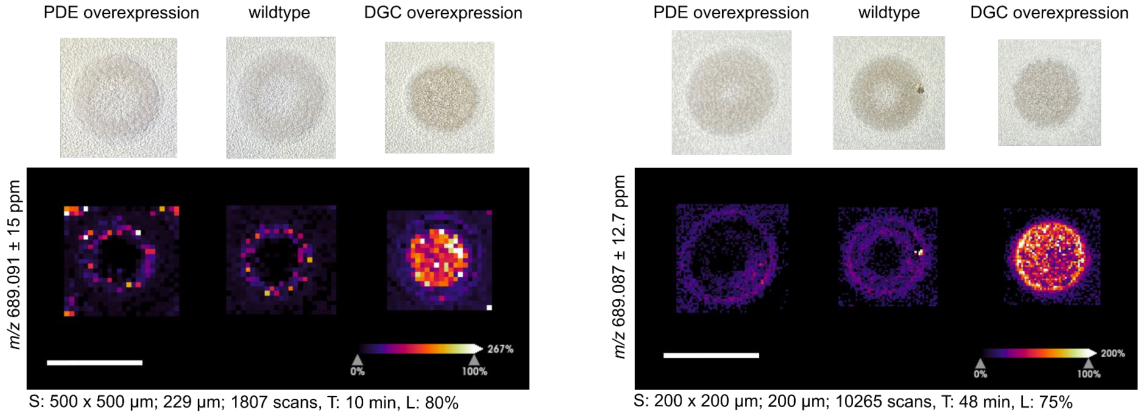

Figure S7. Segmentation analysis for MALDI-MSI on *V. fischeri* colonies shown in Figure 3 using SCiLS Lab. Each color in **a)** represents a segment containing statistically spatially co-localized features within the dataset. **b)** The segmentation tree and the number of features within each segment. The initial two segments (red and yellow) were identified using the Bisecting k-Means method on all features with weak denoising and the Euclidean metric. The segment containing features within the DGC overexpression colony was further segmented twice, resulting in the final image. This highlights that the biofilm rich DGC overexpression mutant has unique signals, one of which is c-di-GMP.

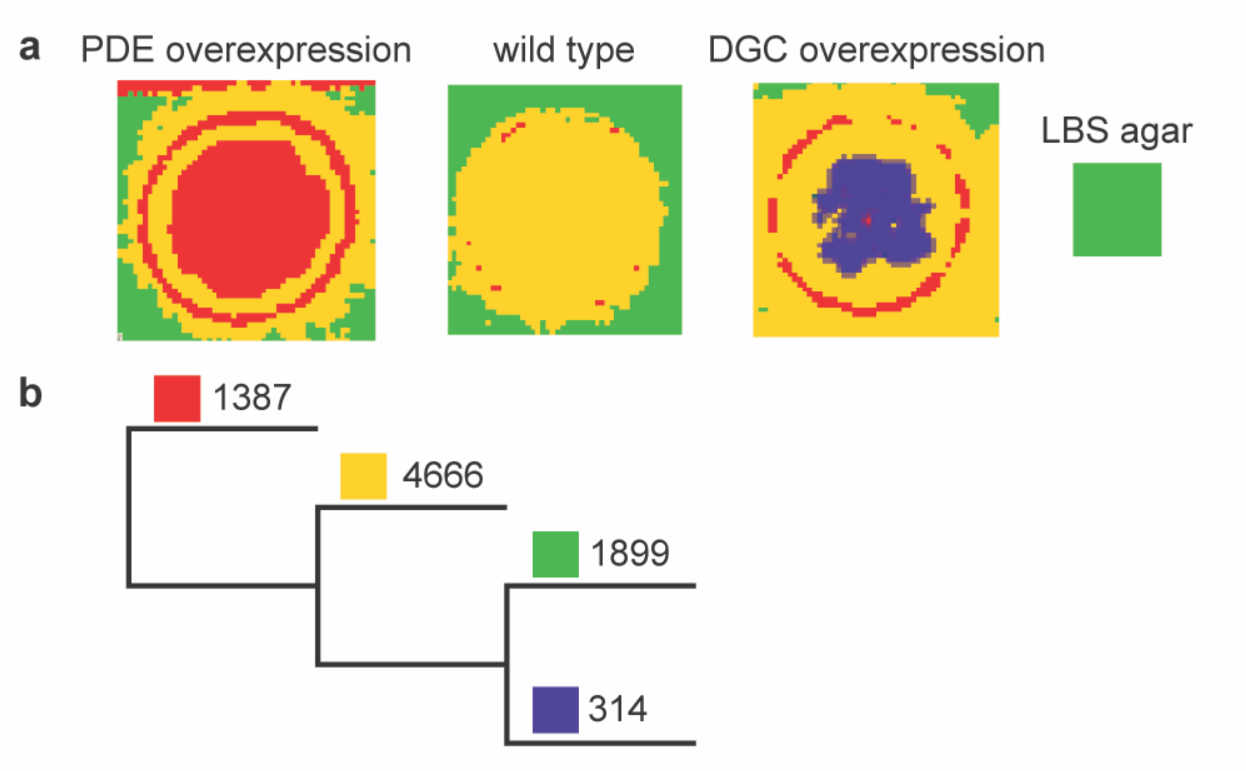

Figure S8. Biological replicate data for MALDI-MSI of *P. aeruginosa* PA14 in Figure 4 showing ion images of c-di-GMP and other putatively identified metabolites in *P. aeruginosa* PA14. Ion images of metabolites detected in **a)** negative mode ionization and **b)** positive mode ionization of one replicate and **c)** negative mode ionization and **d)** positive mode ionization of another replicate. The following compound abbreviations are used: pyocyanin (PYO), phenazine-1-carboxamide (PCN), phenazine-1-carboxylic acid (PCA), *Pseudomonas* quinolone signal (PQS), 4-hydroxy-2-heptyquinoline-N-oxide (HQNO), 2-heptyl-4-quinolone (HHQ), and 4-hydroxy-2-nonylquinoline (HNQ) Table 2 shows the ppm error for all putatively identified compounds. Spot raster; size; scan number (S), acquisition time (T), and laser power (L) shown for each MSI experiment. All scale bars represent 1 cm.

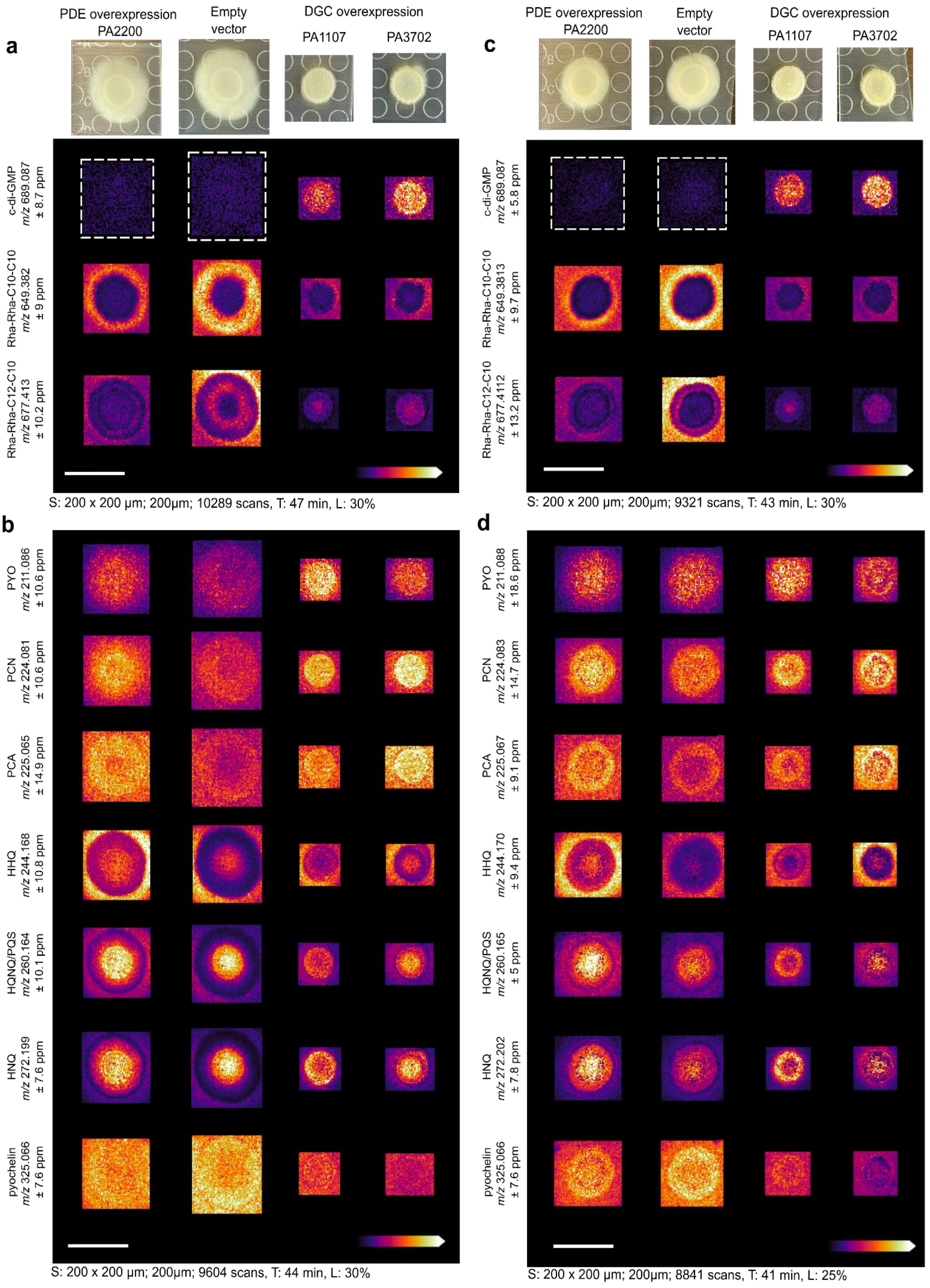

PDE overexpression PA2200
Empty vector
DGC overexpression
PA1107
PA3702
a
c-di-GMP m/z 689.087 ± 8.7 ppm
Rha-Rha-C10-C10 m/z 649.382 ± 9 ppm
Rha-Rha-C12-C10 m/z 677.413 ± 10.2 ppm
S: 200 x 200 µm; 200µm; 10289 scans, T: 47 min, L: 30%
b
PYO m/z 211.086 ± 10.6 ppm
PCN m/z 224.081 ± 10.6 ppm
PCA m/z 225.065 ± 14.9 ppm
HHQ m/z 244.168 ± 10.8 ppm
HQNQ/PQS m/z 260.164 ± 10.1 ppm
HNQ m/z 272.199 ± 7.6 ppm
pyochelin m/z 325.066 ± 7.6 ppm
S: 200 x 200 µm; 200µm; 9604 scans, T: 44 min, L: 30%
c
c-di-GMP m/z 689.087 ± 5.8 ppm
Rha-Rha-C10-C10 m/z 649.3813 ± 9.7 ppm
Rha-Rha-C12-C10 m/z 677.4112 ± 13.2 ppm
S: 200 x 200 µm; 200µm; 9321 scans, T: 43 min, L: 30%
d
PYO m/z 211.088 ± 18.6 ppm
PCN m/z 224.083 ± 14.7 ppm
PCA m/z 225.067 ± 9.1 ppm
HHQ m/z 244.170 ± 9.4 ppm
HQNQ/PQS m/z 260.165 ± 5 ppm
HNQ m/z 272.202 ± 7.8 ppm
pyochelin m/z 325.066 ± 7.6 ppm
S: 200 x 200 µm; 200µm; 8841 scans, T: 41 min, L: 25%

Species	Strain	Genotype	Ref.	Strain Name in Manuscript
V. cholerae	Fy_Vc_1	*Vibrio cholerae* O1 El Tor A1552, wild type, Rifr	2	wildtype
V. cholerae	Fy_Vc_2	*Vibrio cholerae* O1 El Tor A1552, rugose variant, Rifr	2	rugose variant
V. cholerae	Fy_Vc_1745	Fy_Vc_2 ΔvpvC	3	*RΔvpvC*
V. cholerae	FY_VC_11823	FY_VC_1, pFY4950, Rif Gent	this study	wildtype biosensor
V. cholerae	FY_VC_17439	FY_VC_2, pFY4950, Rif Gent	this study	rugose variant biosensor
V. fischeri	MJM2773	MJM1100 / pEVS143-MifA	4	DGC overexpression
V. fischeri	MJM3091	MJM1100 / pEVS143-VF_0087	4	PDE overexpression
V. fischeri	MJM4094	MJM1100 / pRYI039	4	wildtype
P. aeruginosa	PA14 pMMB	PA14 pMMB-gent	5	empty vector
P. aeruginosa	PA14 pMMB PA2200	PA14 pMMB PA2200	5	PDE overexpression
P. aeruginosa	PA14 pMMB PA1107	PA14 pMMB PA1107	5	DGC overexpression
P. aeruginosa	PA14 pMMB PA3702	PA14 pMMB PA3702	5	DGC overexpression

Table S1: Bacterial strains genotype and sources

References

(1) McCaughey, C. S.; Trebino, M. A.; Yildiz, F. H.; Sanchez, L. M. Utilizing Imaging Mass Spectrometry to Analyze Microbial Biofilm Chemical Responses to Exogenous Compounds. *Methods Enzymol.* **2022**, *665*, 281–304.

(2) Beyhan, S.; Yildiz, F. H. Smooth to Rugose Phase Variation in Vibrio Cholerae Can Be Mediated by a Single Nucleotide Change That Targets c-Di-GMP Signalling Pathway. *Mol. Microbiol.* **2007**, *63* (4), 995–1007.

(3) Yildiz, F. H.; Schoolnik, G. K. Vibrio Cholerae O1 El Tor: Identification of a Gene Cluster Required for the Rugose Colony Type, Exopolysaccharide Production, Chlorine Resistance, and Biofilm Formation. *Proc. Natl. Acad. Sci. U. S. A.* **1999**, *96* (7), 4028–4033.

(4) Isenberg, R. Y.; Holschbach, C. S.; Gao, J.; Mandel, M. J. Functional Analysis of Cyclic Diguanylate-Modulating Proteins in Vibrio Fischeri. *bioRxiv* **2023**. https://doi.org/10.1101/2023.07.24.550417.

(5) Kulasakara, H.; Lee, V.; Brencic, A.; Liberati, N.; Urbach, J.; Miyata, S.; Lee, D. G.; Neely, A. N.; Hyodo, M.; Hayakawa, Y.; Ausubel, F. M.; Lory, S. Analysis of Pseudomonas Aeruginosa Diguanylate Cyclases and Phosphodiesterases Reveals a Role for Bis-(3'-5')-Cyclic-GMP in Virulence. *Proc. Natl. Acad. Sci. U. S. A.* **2006**, *103* (8), 2839–2844.
